# EOB-MR Based Radiomics Analysis to Assess Clinical Outcomes following Liver Resection in Colorectal Liver Metastases

**DOI:** 10.3390/cancers14051239

**Published:** 2022-02-27

**Authors:** Vincenza Granata, Roberta Fusco, Federica De Muzio, Carmen Cutolo, Sergio Venanzio Setola, Federica Dell’Aversana, Alessandro Ottaiano, Guglielmo Nasti, Roberta Grassi, Vincenzo Pilone, Vittorio Miele, Maria Chiara Brunese, Fabiana Tatangelo, Francesco Izzo, Antonella Petrillo

**Affiliations:** 1 Division of Radiology, Istituto Nazionale Tumori IRCCS Fondazione Pascale—IRCCS di Napoli, 80131 Napoli, Italy; s.setola@istitutotumori.na.it (S.V.S.); a.petrillo@istitutotumori.na.it (A.P.); 2 Medical Oncology Division, Igea SpA, 41012 Carpi, Italy; r.fusco@igeamedical.com; 3 Diagnostic Imaging Section, Department of Medical and Surgical Sciences & Neurosciences, University of Molise, 86100 Campobasso, Italy; demuziofederica@gmail.com (F.D.M.); m.brunese@studenti.unimol.it (M.C.B.); 4 Department of Medicine, Surgery and Dentistry, University of Salerno, 84084 Fisciano, Italy; carmencutolo@hotmail.it (C.C.); vpilone@unisa.it (V.P.); 5 Division of Radiology, Università degli Studi della Campania Luigi Vanvitelli, 81100 Caserta, Italy; federica.dellaversana@unicampania.it (F.D.); roberta.grassi@policliniconapoli.it (R.G.); 6 Division of Abdominal Oncology, Istituto Nazionale Tumori IRCCS Fondazione Pascale—IRCCS di Napoli, 80131 Napoli, Italy; a.ottaiano@istitutotumori.na.it (A.O.); g.nasti@istitutotumori.na.it (G.N.); 7 Division of Radiology, Azienda Ospedaliera Universitaria Careggi, 50134 Firenze, Italy; vmiele@sirm.org; 8 Italian Society of Medical and Interventional Radiology (SIRM), SIRM Foundation, 20122 Milan, Italy; 9 Division of Pathology, Istituto Nazionale Tumori IRCCS Fondazione Pascale—IRCCS di Napoli, 80131 Napoli, Italy; f.tatangelo@istitutotumori.na.it; 10 Division of Epatobiliary Surgical Oncology, Istituto Nazionale Tumori IRCCS Fondazione Pascale—IRCCS di Napoli, 80131 Napoli, Italy; f.izzo@istitutotumori.na.it

**Keywords:** Liver metastasis, Magnetic Resonance Imaging, artificial intelligence, radiomics

## Abstract

**Simple Summary:**

The aim of this study was to assess the efficacy of radiomics features obtained by EOB-MRI phase in order to predict clinical outcomes following liver resection in Colorectal Liver Metastases Patients, and evaluate recurrence, mutational status, pathological characteristic (mucinous) and surgical resection margin. Ours results confirmed the capacity of radiomics to identify, as biomarkers, several prognostic features that could affect the treatment choice in patients with liver metastases, in order to obtain a more personalized approach. These results were confirmed by external validation dataset. We obtained a good performance considering the single textural significant metric in the identification of front of tumor growth (expansive versus infiltrative) and tumor budding (high grade versus low grade or absent), in the recognition of mucinous type and in the detection of recurrences.

**Abstract:**

The aim of this study was to assess the efficacy of radiomics features obtained by EOB-MRI phase in order to predict clinical outcomes following liver resection in Colorectal Liver Metastases Patients, and evaluate recurrence, mutational status, pathological characteristic (mucinous) and surgical resection margin. This retrospective analysis was approved by the local Ethical Committee board of National Cancer of Naples, IRCCS “Fondazione Pascale”. Radiological databases were interrogated from January 2018 to May 2021 in order to select patients with liver metastases with pathological proof and EOB-MRI study in pre-surgical setting. The cohort of patients included a training set (51 patients with 61 years of median age and 121 liver metastases) and an external validation set (30 patients with single lesion with 60 years of median age). For each segmented volume of interest by 2 expert radiologists, 851 radiomics features were extracted as median values using PyRadiomics. non-parametric test, intraclass correlation, receiver operating characteristic (ROC) analysis, linear regression modelling and pattern recognition methods (support vector machine (SVM), k-nearest neighbors (KNN), artificial neural network (NNET), and decision tree (DT)) were considered. The best predictor to discriminate expansive versus infiltrative front of tumor growth was HLH_glcm_MaximumProbability extraxted on VIBE_FA30 with an accuracy of 84%, a sensitivity of 83%, and a specificity of 82%. The best predictor to discriminate tumor budding was Inverse Variance obtained by the original GLCM matrix extraxted on VIBE_FA30 with an accuracy of 89%, a sensitivity of 96% and a specificity of 65%. The best predictor to differentiate the mucinous type of tumor was the HHL_glszm_ZoneVariance extraxted on VIBE_FA30 with an accuracy of 85%, a sensitivity of 46% and a specificity of 95%. The best predictor to identify tumor recurrence was the LHL_glcm_Correlation extraxted on VIBE_FA30 with an accuracy of 86%, a sensitivity of 52% and a specificity of 97%. The best linear regression model was obtained in the identification of the tumor growth front considering the height textural significant metrics by VIBE_FA10 (an accuracy of 89%; sensitivity of 93% and a specificity of 82%). Considering significant texture metrics tested with pattern recognition approaches, the best performance for each outcome was reached by a KNN in the identification of recurrence with the 3 textural significant features extracted by VIBE_FA10 (AUC of 91%, an accuracy of 93%; sensitivity of 99% and a specificity of 77%). Ours results confirmed the capacity of radiomics to identify as biomarkers, several prognostic features that could affect the treatment choice in patients with liver metastases, in order to obtain a more personalized approach.

## 1. Introduction

Radiomics is a rapidly evolving field of research concerned with the extraction of quantitative metrics—the so-called radiomics features—within medical images. Radiomic features capture tissue and lesion characteristics such as heterogeneity and shape and may, alone or in combination with demographic, histologic, genomic, or proteomic data, be used for clinical problem solving. In oncology, the assessment of tissue heterogeneity is of particular interest; genomic analyses have demonstrated that the degree of tumor heterogeneity is a prognostic determinant of survival and an obstacle to cancer control. Studies have demonstrated that radiomics features are strongly correlated with heterogeneity indices at the cellular level [1,2,3,4,5,6,7,8]. Therefore, that Radiomics could support cancer detection, diagnosis, evaluation of prognosis and response to treatment, so as could supervise disease status [9,10,11,12,13,14]. Using standard of care images that are usually obtained in a clinical setting, Radiomics analysis is a cost-effective and highly feasible implement for clinical decision support, providing prognostic and/or predictive biomarkers which enables a fast, low-cost, and repeatable tool for longitudinal monitoring [15,16,17,18,19,20]. Even though individual features may correlate with genomic data, so-called radiogenomics, or clinical outcomes, the impact of radiomics is increased when the data are processed using machine learning techniques. Nowadays, several studies have assessed the role of radiogenomics in hepatocellular carcinoma, but only a few have examined liver metastases [1,2,3].

During the work-up of patients with liver metastases, imaging plays an important role, since it enables one to estimate the number and sites of lesions, to assess the resectability, and to evaluate the response to treatment and drug toxicities [21,22,23,24,25]. Though computed tomography (CT) is routinely used for primary staging and disease surveillance, Magnetic Resonance imaging (MRI) is a valuable diagnostic technique in oncologic settings since it allows one to assess morphological and functional data [21,22,23,24]. Moreover, several liver-specific contrast agents have been introduced to improve lesions detection and characterization. Gadobenate dimeglumine (Gd-BOPTA) and gadolinium ethoxybenzyl diethylenetriamine pentaacetic acid (Gd-EOB-DTPA) allow one to obtain data on the lesions vascularization during the different phases of contrast study and functional data in the delayed, hepatobiliary phase (EOB-phase).

In this context, the possibility to correlate radiomics parameters obtained by MRI studies to recurrences, mutational status, pathological characteristic (mucinous and tumor budding), and surgical resection margin offers notable advantages over qualitative imaging assessment, allowing a better patient selection for cancer therapy, treatment response prediction, and discrimination of favorable subsets of patients from those with poor prognosis. In the present study, we assessed the efficacy of radiomics features obtained by EOB-MRI phase to predict clinical outcomes following liver resection in Colorectal Liver Metastases Patients. 

## 2. Materials and Methods

### 2.1. Dataset Characteristics

This study aligned with National appropriate guidelines and procedures. The National Cancer Institute of Naples Ethical Committee board approved this retrospective study, renouncing the need for informed patient consent given the study nature.

Radiological archive was evaluated from January 2018 to May 2021 in order to choose patients with: (1) liver metastases with pathological proof; (2) EOB-MRI study in pre-surgical setting after neoadjuvant chemotherapy; (3) MR images of high quality; (4) a follow-up CT scan of at least six months after surgery. The exclusion criteria were: (1) discordance among the imaging diagnosis and the pathologically ones, (2) no EOB-MRI phase studies; (3) no high-quality MR images.

The cohort of patients included a training set and an external validation set. The internal training set consisted of 51 patients (33 women and 18 men) with a median age of 61 years (range 35–82 years) and 121 liver metastases. The validation cohort consisted of 30 patients with single lesion (10 women and 20 men) with 60 years of median age (range 40–78 years). The external validation patient dataset was provided by “Careggi Hospital”, Florence, Italy.

No liver metastases identified by EOB-MRI during the period investigated in this retrospective study were pathologically confirmed and thus not included in the study.

The patient characteristics are summarized in Table 1.

### 2.2. MR Imaging Protocol

MR studies were performed with 2 1.5T MR tomographs: a Magnetom Symphony (Siemens, Erlangen, Germany) and Magnetom Aera (Siemens). The MRI images were acquired before and after an intravenous (IV) contrast agent (CA) injection.

In this study, a radiomics features extraction was made on volumetric interpolated breath-hold examination (VIBE) T1-weighted SPAIR with controlled respiration used to acquire images after IV CA injection with a liver-specific CA (0.1 mL/kg of Gd-EOB-BPTA—Primovist, Bayer Schering Pharma, Berlin, Germany). The VIBE T1-W sequence was acquired with 2 different flip angles (10 and 30 degrees). A power injector (Spectris Solaris^®^ EP MR, MEDRAD Inc., Indianola, IA, USA) was used to administrate the CA at an infusion rate of 2 mL/s, as descripted in our previous studies [26,27]. Table 2 reports MR Sequence parameters.

### 2.3. Image Processing

Regions of interest (ROIs) were manually drawn slice-by-slice by 2 expert radiologists with 22 and 15 years of abdominal imaging experience, respectively; first separately and then together and in accordance with each other, annotating all of the slices of the lesions. The segmentation was performed on arterial phase of VIBE T1-W images for both sequences acquired using 10 and 30 degrees of flip angle. For these reasons, we performed the analysis on 2 sequence VIBE_FA10 (VIBE T1-W images with flip angle 10°) and VIBE_FA30 (VIBE T1-W images with flip angle 30°). Manual definition of the ROIs was made using segmentation tool of 3DSlicer (https://www.slicer.org/; accessed on 21 December 2021).

### 2.4. MRI Post-Processing with Pyradiomics Tool

For each volume of interest, 851 radiomics features were extracted as median values using open-source PyRadiomics python package [28].

We used wavelet filtering (LLH, LHL, LHH, LLL, HLL, HLH, HHL, HHH high (H) or low (L) -pass filters along the X and Y axis and a *Z*-axis) to six different matrices:

- First Order (FIRST ORDER): describes the individual values of voxels obtained as a result of ROI cropping. These are generally histogram-based properties (energy, entropy, kurtosis, skewness);
- Shape based features both 2D and 3D measures;
- Gray Level Co-occurrence Matrix (GLCM): calculates how often the same and similar pixel values come together in an image and records statistical measurements according to this matrix. These resulting values numerically characterize the texture of the image;
- Gray Level Run Length Matrix (GLRLM): Defined as the number of homogeneous consecutive pixels with the same gray tone and quantifies the gray-level studies;
- Gray Level Size Zone Matrix (GLSZM): Properties based on this matrix assign voxel counts according to the logic of measuring gray-level regions in an image;
- Neighboring Gray Tone Difference Matrix (NGTDM): Digitization of textures obtained from filtered images and their fractal properties;
- Gray Level Dependence Matrix (GLDM): Number of bound voxels at x distance from the central voxel;

The extracted features are in compliance with feature definitions as described by the Imaging Biomarker Standardization Initiative (IBSI) [29] and as reported in (https://readthedocs.org/projects/pyradiomics/downloads/; accessed on 21 December 2021).

Median values of radiomics features were considered for each segmented volume of interest.

**Figure 1:**
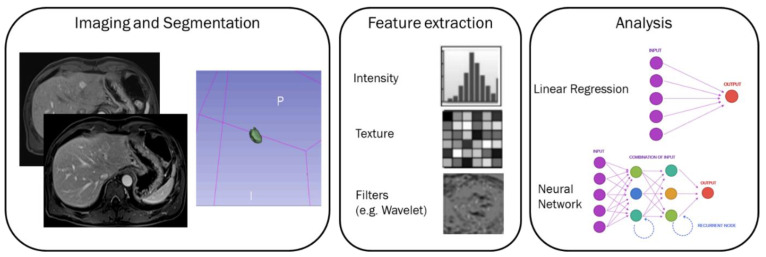
A graphical representation of the radiomics process and of the extracted features.

A graphical representation of the radiomics process and of the extracted features has been reported in Figure 1. However, Radiomics involves 3D qualitative and quantitative high throughput extraction of digital imaging data that cannot be represented as an image.

Radiomics analysis was performed blinded to the clinical and pathological data in pre-surgical setting after neoadjuvant chemotherapy. No registration techniques to reduce movements artefacts were applied, however, the use of median value of extracted metrics allows one to reduce the influence of artefacts. 

### 2.5. Statistical Analysis

Statistical analysis includes both univariate and multivariate approaches performed considering a per-lesion analysis. The statistical analyses were performed using the Statistics and Machine Toolbox of MATLAB R2021b (MathWorks, Natick, MA, USA).

### 2.6. Univariate Analysis

The assessment of observer variability was performed by calculating the intraclass correlation coefficient.

A non-parametric Kruskal-Wallis test was performed to identify differences statistically significant among clinical parameters and radiomics metrics of two groups (front of tumor growth: expansive versus infiltrative; tumor budding: high grade versus low grade or absent; mucinous type and presence of recurrence).

Receiver operating characteristic (ROC) analysis was performed using the Youden index to calculate the optimal cut-off for each metric and then the area under the ROC curve (AUC), sensitivity, specificity, positive predictive value (PPV), negative predictive value (NPV) and accuracy.

A *p* value < 0.05 was considered as significant.

### 2.7. Multivariate Analysis

A multivariate analysis was performed in order to identify the combinations of variables which best predict the outcomes: (1) front of tumor growth: expansive versus infiltrative; (2) tumor budding: high grade versus low grade or absent; (3) mucinous type; (4) presence of recurrence.

Given the high number of textural features, a first selection of variables was made based on the results obtained from the univariate analysis: significant at nonparametric Kruskal-Wallis test and with an accuracy ≥ 75%. A linear regression modelling was used to assess the best linear combination of significant textural features for each outcome. The linear regression model was used to assess the accuracy of linear combination and ROC analysis with Youden index was used to identify the optimal cut-off value. Considering the optimal cut-off value, we reported accuracy, sensitivity, specificity, PPV and NPV.

Pattern recognition techniques including support vector machine (SVM), k-nearest neighbors (KNN), artificial neural network (NNET), and decision tree (DT) were performed to calculate the diagnostic performance considering all of the features and/or a subset of features after a feature selection approach [30]. The best model was identified calculating the highest area under ROC curve and highest accuracy. Each classifier was trained with a 10-k fold cross validation; therefore, median values of AUC, accuracy, sensitivity, and specificity were calculated. Moreover, an external validation cohort was used to validate the findings of the best classifier.

## 3. Results

### 3.1. Univariate Analysis Findings

The median value of intraclass correlation coefficients for features was 0.92 (range 0.86–0.96). The size of the lesion did not affect the extracted metrics (*p*-value > 0.05 at the Kruskal-Wallis test performed between the 2 groups: lesions < 2 cm and lesions ≥ 2 cm). In addition, the RAS mutational status did not affect the extracted metrics (*p*-value > 0.05 at the Kruskal-Wallis test performed between the groups). Therefore, considering homogeneous the two groups respect to the extracted radiomics metrics, RAS mutational was not considered for the following analysis.

There were no differences between the extracted radiomics metrics on VIBE_FA10 and on VIBE_FA30 (*p*-value > 0.05 at the Kruskal-Wallis test).

Significant radiomics metrics for each outcome at univariate analysis are reported in Table 3.

Among the significant features to differentiate the front of tumor growth on VIBE_FA10, 8 textural parameters obtained an accuracy ≥ 75%. Among these 8 features, the best predictor to discriminate expansive versus infiltrative front of tumor growth was HHL_glcm_MaximumProbability with an accuracy of 81%, a sensitivity of 92%, a specificity of 62%, a PPV and a NPV of 80% and 82%, respectively.

Among the significant features to differentiate the front of tumor growth on VIBE_FA30, 15 textural parameters obtained an accuracy ≥ 75%. Among these 15 features, the best predictor to discriminate expansive versus infiltrative front of tumor growth was HLH_glcm_MaximumProbability (the same feature of previous case obtained with another wavelet filter HLH respect to HHL) with an accuracy of 84%, a sensitivity of 83%, a specificity of 82%, a PPV and a NPV of 89% and 74%, respectively.

Among the significant features to differentiate the tumor budding on VIBE_FA10, 8 textural parameters obtained an accuracy ≥ 85%. Among these 8 features, the best predictor to discriminate tumor budding was again the HHL_glcm_MaximumProbability with an accuracy of 88%, a sensitivity of 94%, a specificity of 68%, a PPV and a NPV of 89% and 81%, respectively.

Among the significant features to differentiate the tumor budding on VIBE_FA30, 16 textural parameters obtained an accuracy ≥ 85%. Among these 16 features, the best predictor to discriminate tumor budding was Inverse Variance obtained by the original GLCM matrix with an accuracy of 89%, a sensitivity of 96%, a specificity of 65%, a PPV and a NPV of 89% and 83%, respectively.

Among the significant features to differentiate the mucinous type of tumor on VIBE_FA10, 8 textural parameters obtained an accuracy ≥ 80%. Among these 8 features, the best predictor to differentiate the mucinous type of tumor was the HHH_ngtdm_Busyness with an accuracy of 84%, a sensitivity of 65%, a specificity of 42%, a PPV and a NPV of 69% and 86%, respectively.

Among the significant features to differentiate the mucinous type of tumor on VIBE_FA30, 12 textural parameters obtained an accuracy ≥ 80%. Among these 12 features, the best predictor to differentiate the mucinous type of tumor was the HHL_glszm_ZoneVariance with an accuracy of 85%, a sensitivity of 46%, a specificity of 95%, a PPV and a NPV of 71% and 87%, respectively.

Among the significant features to identify tumor recurrence on VIBE_FA10, 3 textural parameters obtained an accuracy ≥ 80%. Among these 3 features, the best predictor to identify tumor recurrence was the LLH_glrlm_ShortRunEmphasis with accuracy of 85%, a sensitivity of 31%, a specificity of 100%, a PPV and a NPV of 100% and 84%, respectively.

Among the significant features to identify tumor recurrence on VIBE_FA30, 8 textural parameters obtained an accuracy ≥ 80%. Among these 8 features, the best predictor to identify tumor recurrence was the LHL_glcm_Correlation with an accuracy of 86%, a sensitivity of 52%, a specificity of 97%, a PPV and a NPV of 84% and 85%, respectively.

**Figure 2:**
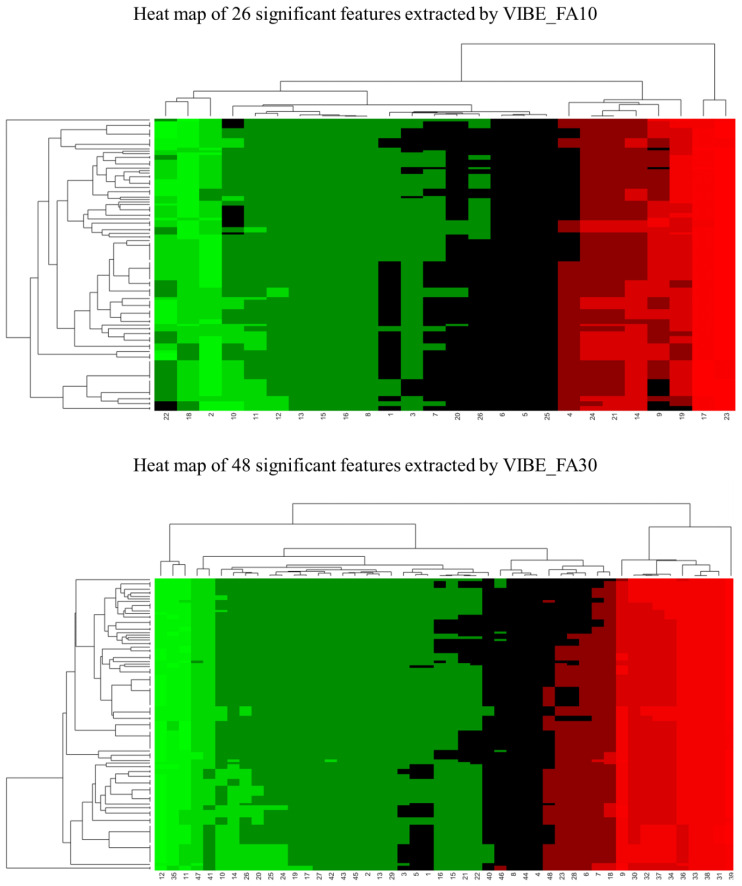
Heat maps pf significant radiomics features (26 extracted by VIBE_FA10 and 48 extracted by VIBE_FA30).

In total, 26 features extracted by VIBE_FA10 were resulted significant at univariate analysis while 48 were resulted significand among those extracted on VIBE_FA30. Figure 2 shows a heat map.

### 3.2. Multivariate Analysis Findings

#### 3.2.1. Linear Regression Analysis Findings

**Figure 3:**
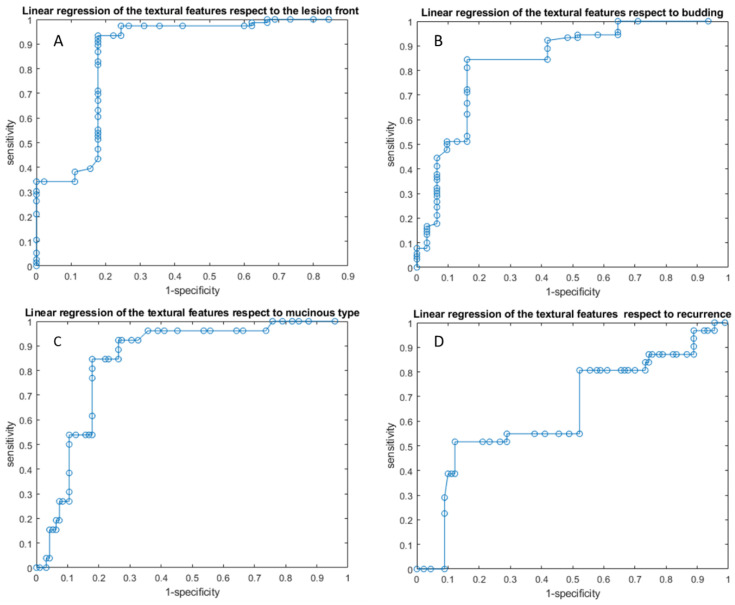
ROC curves of linear regression analysis respect to the front of tumor growth (**A**), the tumor budding (**B**), the tumor mucinous type (**C**), recurrence presence (**D**) obtained considering significant features extracted by VIBE_FA10.

**Figure 4:**
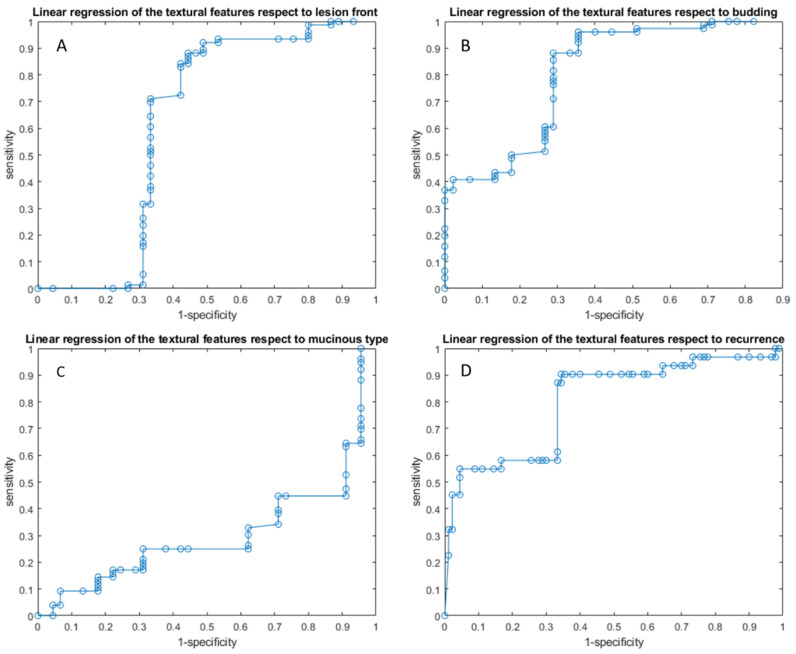
ROC curves of linear regression analysis respect to the front of tumor growth (**A**), the tumor budding (**B**), the tumor mucinous type (**C**), recurrence presence (**D**) obtained considering significant features extracted by VIBE_FA30.

Linear regression models obtained good results in each considered classification problem (1. Front of tumor growth: expansive versus infiltrative; 2. tumor budding: high grade versus low grade or absent; 3. mucinous type; 4. presence of recurrence) with an accuracy in the range of 72 to 89% Table 4 and Table 5, Figure 3 and Figure 4. The best linear regression model was obtained in the identification of the front of tumor growth considering the height textural significant metrics by VIBE_FA10 (AUC of 72%, an accuracy of 89%; sensitivity of 93% and a specificity of 82%). The coefficients of this linear models are reported in the Table 6.

#### 3.2.2. Pattern Recognition Approaches Findings

**Figure 5:**
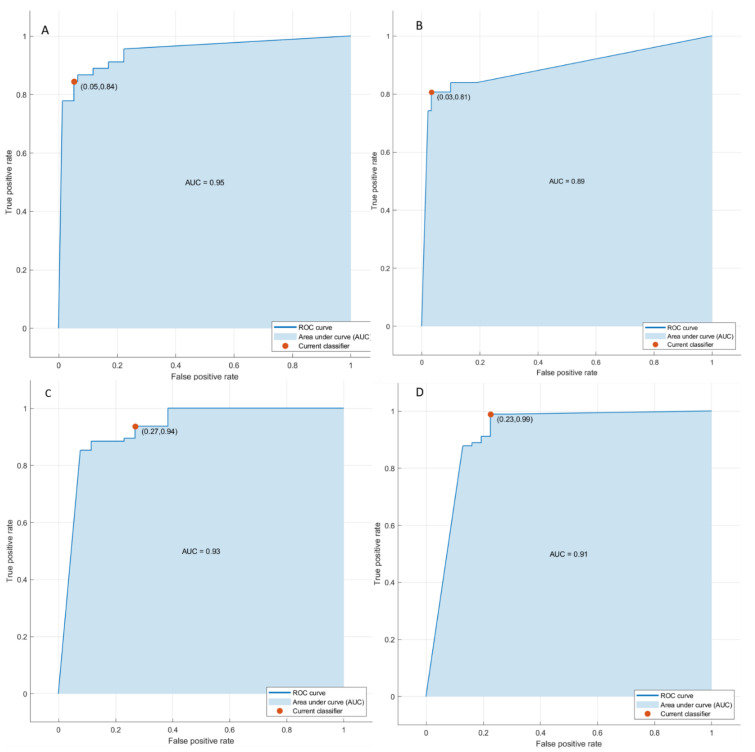
ROC curves of KNN respect to the front of tumor growth (**A**), the tumor budding (**B**), the tumor mucinous type (**C**), recurrence presence (**D**) obtained considering significant features extracted by VIBE_FA10.

**Figure 6:**
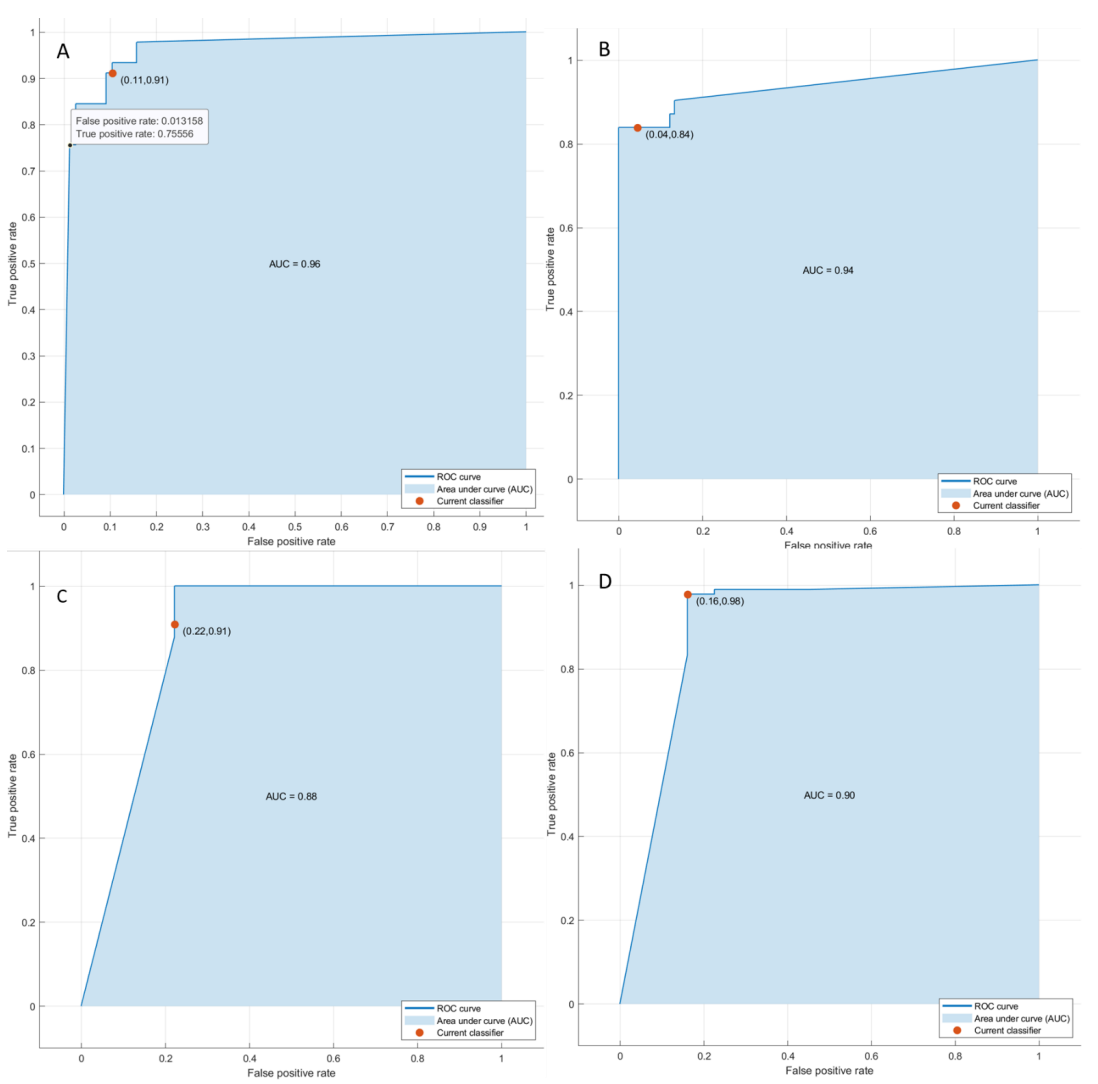
ROC curves of KNN respect to the front of tumor growth (**A**), the tumor budding (**B**), the tumor mucinous type (**C**), recurrence presence (**D**) obtained considering significant features extracted by VIBE_FA30.

Considering the significant texture metrics tested with pattern recognition approaches, the best performance for each outcome (1. Front of tumor growth: expansive versus infiltrative; 2. tumor budding: high grade versus low grade or absent; 3. mucinous type and 4. presence of recurrence) was reached by a KNN, both considering the features extracted by VIBE_FA10 and VIBE_FA30. The accuracy was always major to 88% (Table 4 and Table 5, Figure 5 and Figure 6) both on training and validation set and the best results was obtained in the identification of recurrence with the 3 textural significant features extracted by VIBE_FA10 (AUC of 91%, an accuracy of 93%; sensitivity of 99% and a specificity of 77%).

## 4. Discussions

Ours results confirmed the capacity of radiomics to identify as biomarkers, several prognostic features that could affect the treatment choice in patients with liver metastases, in order to obtain a more personalized approach. In fact, the possibility to correlate radiomics parameters to RAS status offers notable advantages over qualitative imaging assessment, allowing one to tailor cancer therapy to the patient, to predict response to treatment, to distinguish favorable subsets of patients from those with poor prognosis, to select patients that may benefit of surgical treatment. Literature data underlines the role of several features, as RAS mutation, front of tumor growth, tumor budding and mucinous type as a strong prognostic and predictive biomarker in patients subjected to target therapy or surgical resection. In this scenario, our results confirmed the possibility of radiomics to allow one to tailor cancer therapy at the patient, to predict response to treatment, to detect favorable subsets of patients from those with poor prognosis and to select patients that may benefit from surgical treatment [5,6].

Our results were confirmed by external validation dataset. We obtained a good performance considering the single textural significant metric in the identification of front of tumor growth (expansive versus infiltrative) and tumor budding (high grade versus low grade or absent), in the recognition of mucinous type and in the detection of recurrences. The median value of intraclass correlation coefficients for features was 0.92.

With regard to the front of tumor growth on VIBE_FA10, the best performance was obtained with HHL_glcm_MaximumProbability with an accuracy of 81%; while on VIBE_FA30, the best performance was with HLH_glcm_MaximumProbability (the same feature of previous case obtained with another wavelet filter HLH respect to HHL) with an accuracy of 84%. Among significant features to differentiate the tumor budding on VIBE_FA10, the best predictot was again the HHL_glcm_MaximumProbability with an accuracy of 88% while on VIBE_FA30, the best performance was of the Inverse Variance extracted by the original GLCM matrix with an accuracy of 89%.

Regarding to differentiate the mucinous type of tumor on VIBE_FA10, the best predictor was the HHH_ngtdm_Busyness with an accuracy of 84% while on VIBE_FA30, the best performance was obtained by the HHL_glszm_ZoneVariance with an accuracy of 85%.

Among the significant features to identify tumor recurrence on VIBE_FA10, the best performance was obtained by the LLH_glrlm_ShortRunEmphasis with an accuracy of 85% while on VIBE_FA30, the best predictor was the LHL_glcm_Correlation with an accuracy of 86%.

Therefore, all of the significant and better predictors for each outcome except that the Inverse Variance obtained by the original GLCM matrix were Higher-order statistics features obtained by statistical methods after wavelet transform. However, all of these metrics capture certain statistical regularities of tumor lesions through images linked to the heterogeneity of gray levels on the segmented volume of interest.

Considering a linear regression models or neural network classifiers in a multivariate approach was possible to increase the performance in terms of accuracy, sensitivity, and specificity. The best linear regression model was obtained in the identification of the front of tumor growth considering the height textural significant metrics by VIBE_FA10 (AUC of 72%, an accuracy of 89%; sensitivity of 93% and a specificity of 82%) while the best results with a KNN was obtained in the identification of recurrence with the 3 textural significant features (AUC of 91%, an accuracy of 93%; sensitivity of 99% and a specificity of 77%).

Research has shown the association between entropy and prognosis [31,32,33,34,35,36,37,38,39,40]. Homogeneity in the texture of healthy liver tissue is predictive of worse survival. Andersen et al. [32] demonstrated a link between homogeneity features and overall survival (OS). Rahmim et al. [37] showed as radiomics data of heterogeneity, obtained by FDG PET study, were prognosticators of lower OS [37]. Research has shown the degree of skewness was inversely correlated with KRAS status, while the entropy was related to OS [34]. Moreover, the opportunity to identify the patients for recurrence has been shown [37,38,39,40,41]. Ravanelli et al. [39] correlated high CT uniformity and low OS and Progression Free Survival (PFS).

Radiomics and radiogenomics are emerging fields with important weaknesses that need to be taken into account. The main limit is the heterogeneity of software analysis and the variety of the metrics in different hospitals. Therefore, the segmentation of lesion may affect results [41].

The present study had several limitations: (1) the small population size considered, although the analysis was conducted on a homogeneous sample and on all individual lesions; (2) the retrospective nature of the study, (3) a manual segmentation, that, although research has supported automatic segmentation to avoid inter-observer variability, in our opinion, the manual approach is more realistic. Moreover, we did not assess the impact of the contrast administration and the different phases of contrast study (arterial, portal and transitional phase) respect to EOB-phase, data that we are evaluating in a future paper. However, we evaluated the impact of different flip angle (10 and 30). Additionally, we did not evaluate the impact of chemotherapy on our data.

## 5. Conclusions

Ours results confirmed the capacity of radiomics to identify, as biomarkers, several prognostic features that could affect the treatment choice in patients with liver metastases, in order to obtain a more personalized approach. These results were confirmed by external validation dataset. We obtained a good performance considering the single textural significant metric in the identification of front of tumor growth (expansive versus infiltrative) and tumor budding (high grade versus low grade or absent), in the recognition of mucinous type and in the detection of recurrences.

## Figures and Tables

**Table 1:** Characteristics of the study population (81 patients).

Patient Description	Numbers (%)/Range
Gender	Men 53 (65.4%)
	Women 28 (34.6%)
Age	61 years; range: 35–82 years
**Primary Cancer Site**	
Colon	52 (64.2%)
Rectum	29 (35.8%)
**Prior Chemotherapy**	81 (100%)
**Hepatic Metastases Description**	
Patients with single nodule	52 (64.2%)
Patients with multiple nodules	29 (35.8%)/range: 2–13 metastases
Nodule size (mm)	mean size 36.4 mm; range 7–58 mm
**Front of Tumor Growth**	
expansive	30 (37.0%)
infiltrative	51 (63.0%)
**Tumor Budding**	
Absent	12 (14.8%)
Low grade	14 (17.3%)
High grade	55 (67.9%)
**Mucinous Carcinoma**	25 (30.9%)
**Recurrence**	19 (23.5%)
**RAS Mutation**	42 (51.9%)

**Table 2:** MR Sequence parameters.

Sequence	Orientation	TR/TE/FA (ms/ms/deg.)	AT (min)	Acquisition Matrix	ST/Gap (mm)	FS
Trufisp T2-W	Coronal	4.30/2.15/80	0.46	512 × 512	4/0	without
HASTE T2-W	Axial	1500/90/170	0.36	320 × 320	5/0	without and with (SPAIR)
HASTE T2w	Coronal	1500/92/170	0.38	320 × 320	5/0	without
In-Out phase T1-W	Axial	160/2.35/70	0.33	256 × 192	5/0	without
VIBE T1-W_FA10	Axial	4.80/1.76/10	0.18	320 × 260	3/0	with (SPAIR)
VIBE T1-W_FA30	Axial	4.80/1.76/30	0.18	320 × 260	3/0	with (SPAIR)

Note: W = Weighted, TR = Repetition time, TE = Echo time, FA = Flip angle, AT = Acquisition time, SPAIR = Spectral Adiabatic Inversion Recovery, VIBE = Volumetric interpolated breath hold examination, HASTE = HASTE = Half-Fourier-Acquired Single-shot Turbo spin Echo.

**Table 3:** Significant radiomics features for each considered outcome.

Significant Textural Features Extracted by	VIBE_FA10 Respect to the Front of Tumor Growth	VIBE_FA30 Respect to the Front of Tumor Growth	VIBE_FA10 Respect to the Tumor Budding	VIBE_FA30 Respect to the Tumor Budding	VIBE_FA10 Respect to the Mucinous Type	VIBE_FA30 Respect to the Mucinous Type	VIBE_FA10 Respect to Recurrence	VIBE_FA30 Respect to Recurrence
	Wavelet_HHL_glcm_MaximumProbability	Wavelet_HLH_glcm_MaximumProbability	Wavelet_HHL_glcm_MaximumProbability	Original_glcm_InverseVariance	Wavelet_HHH_ngtdm_Busyness	Wavelet_HHL_glszm_ZoneVariance	Wavelet_LLH_glrlm_ShortRunEmphasis	Wavelet_LHL_glcm_Correlation
AUC	0.66	0.68	0.70	0.70	0.65	0.63	0.48	0.74
Sensitivity	0.92	0.83	0.94	0.96	0.42	0.46	0.31	0.52
Specificity	0.62	0.82	0.68	0.65	0.95	0.96	1.00	0.97
PPV	0.80	0.89	0.89	0.89	0.69	0.75	1.00	0.84
NPV	0.82	0.74	0.81	0.83	0.86	0.87	0.84	0.85
Accuracy	0.81	0.84	0.88	0.89	0.84	0.85	0.85	0.86
Cut-off	0.28	0.28	0.28	0.35	1306.26	1,289,504.66	0.84	0.46

Note: GLCM, Gray Level Co-occurrence Matrix; GLSZM, Gray Level Size Zone Matrix; GLRLM, Gray Level Run Length Matrix; GLDM, Gray Level Dependence Matrix; NGTDM, Neighboring Gray Tone Difference Matrix.

**Table 4:** Linear regression and Pattern recognition analysis with significant features from the VIBE_FA10.

**Linear Regression of Significant Features**	**AUC**	**Sensitivity**	**Specificity**	**PPV**	**NPV**	**Accuracy**	**Cut-Off**
Linear regression of the textural features extracted from the VIBE_FA10 with respect to the front of tumor growth	0.72	0.93	0.82	0.90	0.88	0.89	1.49
Linear regression of the textural features extracted from the VIBE_FA10 with respect to the tumor budding	0.78	0.84	0.84	0.94	0.65	0.84	1.54
Linear regression of the textural features extracted from the VIBE_FA10 with respect to the mucinous type	0.80	0.85	0.82	0.56	0.95	0.83	0.28
Linear regression of the textural features extracted from the VIBE_FA10 with respect to the recurrence presence	0.63	0.52	0.88	0.59	0.84	0.79	3.81
**Pattern Recognition Analysis with Significant Features**	**Dataset**	**AUC**	**Accuracy**	**Sensitivity**	**Specificity**	**Training** **Time [sec]**	**Model Type and Parameters**
KNN	Training set	0.96	0.91	0.84	0.95	8.7	Weighted KNN; number of neighbors:10; distance metric: Euclidean; distance weight: squared inverse
	Validation set	0.97	0.92	1	0.86		
	Training set	0.89	0.93	0.81	0.97	3.9	
	Validation set	0.9	0.93	0.73	1		
	Training set	0.93	0.89	0.94	0.73	3.2	
	Validation set	0.95	0.88	0.91	0.8		
	Training set	0.91	0.93	0.99	0.77	9.21	
	Validation set	0.97	0.94	0.9	0.91		

**Table 5:** Linear regression and Pattern recognition analysis with significant features from the VIBE_FA30.

**Linear Regression of Significant Features**	**AUC**	**Sensitivity**	**Specificity**	**PPV**	**NPV**	**Accuracy**	**Cut-Off**
Linear regression of the textural features extracted from the VIBE_FA30 with respect to the front of tumor growth	0.55	0.88	0.56	0.77	0.74	0.76	8.81
Linear regression of the textural features extracted from the VIBE_FA30 with respect to the tumor budding	0.65	0.96	0.64	0.82	0.91	0.84	0.56
Linear regression of the textural features extracted from the VIBE_FA30 with respect to the mucinous type	0.26	1.00	0.04	0.64	1.00	0.64	−0.17
Linear regression of the textural features extracted from the VIBE_FA30 with respect to the recurrence presence	0.79	0.90	0.66	0.47	0.95	0.72	0.27
**Pattern Recognition Analysis with Significant Features**	**Dataset**	**AUC**	**Accuracy**	**Sensitivity**	**Specificity**	**Training** **time [sec]**	**Model Type and Parameters**
KNN	Training set	0.96	0.90	0.91	0.89	13.4	Weighted KNN; number of neighbors:10; distance metric: Euclidean; distance weight: squared inverse
	Validation set	0.95	0.80	0.67	1		
	Training set	0.94	0.93	0.84	0.96	8.3	
	Validation set	0.94	0.89	0.89	0.89		
	Training set	0.93	0.91	0.96	0.73	7.51	
	Validation set	0.89	0.88	0.89	0.8		
	Training set	0.9	0.94	0.98	0.84	8.4	
	Validation set	0.85	0.91	0.94	0.8		

**Table 6:** Linear regression model parameters.

**Linear Regression of the Textural Features Extracted by VIBE_FA10 with Respect to the Front of Tumor Growth**	** Coefficients **	*** p *** ** Value **	*** p *** ** Value **
Intercept	−7.37	0.08	0.000
original_shape_SurfaceVolumeRatio	−0.85	0.58	
wavelet_LHL_glszm_SmallAreaLowGrayLevelEmphasis	1.50	0.19	
wavelet_LLH_glcm_InverseVariance	4.15	0.00	
wavelet_HHH_glrlm_ShortRunHighGrayLevelEmphasis	0.14	0.00	
wavelet_HHH_glrlm_ShortRunEmphasis	6.25	0.51	
wavelet_HHH_glrlm_RunPercentage	−6.73	0.57	
wavelet_HHH_glrlm_RunLengthNonUniformityNormalized	5.15	0.40	
wavelet_HHL_glcm_MaximumProbability	16.11	0.00	
**Linear Regression of the Textural Features Extracted** **by** **VIBE_FA10 with Respect to the Tumor Budding**	** Coefficients **	*** p *** ** value **	*** p *** ** value **
Intercept	−17.88	0.00	0.000
wavelet_HLL_gldm_LargeDependenceLowGrayLevelEmphasis	0.11	0.32	
wavelet_HLL_glrlm_LongRunLowGrayLevelEmphasis	−3.28	0.33	
wavelet_HLL_glszm_GrayLevelNonUniformityNormalized	−4.28	0.05	
wavelet_LLH_glrlm_GrayLevelNonUniformityNormalized	2.15	0.07	
wavelet_HLH_glcm_JointEnergy	48.41	0.00	
wavelet_HLH_firstorder_10Percentile	0.00	0.96	
wavelet_HHH_glcm_MaximumProbability	1.42	0.87	
wavelet_HHL_glcm_MaximumProbability	24.94	0.00	
**Linear Regression of the Textural Features Extracted** **by** **VIBE_FA10 with Respect to the Mucinous Type**	** Coefficients **	*** p *** ** value **	*** p *** ** value **
Intercept	3.31	0.14	0.000
wavelet_LHL_gldm_DependenceNonUniformity	0.00	0.03	
wavelet_LHL_ngtdm_Strength	−1.20	0.08	
wavelet_LHL_ngtdm_Busyness	0.00	0.07	
wavelet_LHH_glcm_ClusterTendency	7.50	0.00	
wavelet_HLH_gldm_DependenceEntropy	−0.03	0.97	
wavelet_HLH_firstorder_Mean	0.12	0.59	
wavelet_HHH_ngtdm_Busyness	0.00	0.08	
wavelet_HHL_gldm_DependenceEntropy	−1.44	0.04	
**Linear Regression of the Textural Features Extracted** **by** **VIBE_FA10 with Respect to the Recurrence Presence**	** Coefficients **	*** p *** ** value **	*** p *** ** value **
Intercept	−2.95	0.05	0.030
wavelet_LLH_glrlm_ShortRunEmphasis	8.19	0.04	
wavelet_LLH_glrlm_RunLengthNonUniformityNormalized	−5.25	0.10	
wavelet_HHH_ngtdm_Busyness	0.00	0.49	
**Linear Regression of the Textural Features Extracted** **by** **VIBE_FA30 with Respect to the Front of Tumor Growth**	** Coefficients **	*** p *** ** value **	*** p *** ** value **
Intercept	−4.868	0.010	0.000
original_glcm_InverseVariance	1.484	0.260	
wavelet_HLL_gldm_GrayLevelVariance	−4.907	0.206	
wavelet_HLL_glcm_InverseVariance	4.257	0.165	
wavelet_HLL_glcm_DifferenceVariance	−0.967	0.182	
wavelet_HLL_glcm_SumEntropy	1.329	0.195	
wavelet_HLL_glcm_SumSquares	−0.462	0.540	
wavelet_HLL_firstorder_RobustMeanAbsoluteDeviation	0.360	0.244	
wavelet_HLL_firstorder_MeanAbsoluteDeviation	−0.612	0.161	
wavelet_HLL_firstorder_RootMeanSquared	0.153	0.273	
wavelet_HLL_firstorder_RootMeanSquared	0.823	0.717	
wavelet_HLL_firstorder_Variance	0.010	0.162	
wavelet_LHH_glrlm_ShortRunLowGrayLevelEmphasis	−0.018	0.944	
wavelet_HLH_glcm_MaximumProbability	8.613	0.051	
wavelet_HLH_glcm_MaximumProbability	0.903	0.817	
wavelet_LLL_gldm_SmallDependenceLowGrayLevelEmphasis	24.740	0.440	
**Linear Regression of the Textural Features Extracted** **by** **VIBE_FA30 with Respect to the Tumor Budding**	** Coefficients **	*** p *** ** value **	*** p *** ** value **
Intercept	5.220	0.521	0.000
original_glcm_InverseVariance	2.978	0.008	
wavelet_HLL_glcm_JointEnergy	−12.594	0.008	
wavelet_HLL_glcm_Idm	114.999	0.000	
wavelet_HLL_glcm_Id	−123.518	0.002	
wavelet_HLL_firstorder_Uniformity	−18.651	0.011	
wavelet_HLL_firstorder_10Percentile	−0.005	0.801	
wavelet_HLL_glrlm_GrayLevelNonUniformityNormalized	14.712	0.009	
wavelet_HLL_glszm_GrayLevelNonUniformityNormalized	−0.804	0.458	
wavelet_LHL_glcm_Idm	−71.494	0.012	
wavelet_LHL_glcm_Id	79.815	0.014	
wavelet_LHH_firstorder_10Percentile	−0.003	0.941	
wavelet_LLH_firstorder_Uniformity	10.540	0.002	
wavelet_LLH_glrlm_GrayLevelNonUniformityNormalized	−12.808	0.002	
wavelet_LLH_glszm_GrayLevelNonUniformityNormalized	2.066	0.156	
wavelet_HHL_glcm_JointEnergy	1.672	0.853	
wavelet_HHL_firstorder_10Percentile	0.202	0.006	
**Linear Regression of the Textural Features Extracted** **by** **VIBE_FA30 with Respect to the Mucinous Type**	** Coefficients **	*** p *** ** value **	*** p *** ** value **
Intercept	13.293	0.018	0.000
original_shape_SurfaceVolumeRatio	−2.669	0.000	
wavelet_LHH_gldm_DependenceNonUniformity	0.004	0.113	
wavelet_LHH_gldm_GrayLevelNonUniformity	0.000	0.710	
wavelet_HLH_gldm_DependenceNonUniformity	−0.014	0.000	
wavelet_HLH_glrlm_GrayLevelNonUniformity	0.005	0.003	
wavelet_HHH_gldm_DependenceNonUniformity	−0.002	0.473	
wavelet_HHH_glszm_ZonePercentage	67.121	0.000	
wavelet_HHH_ngtdm_Busyness	0.000	0.000	
wavelet_HHL_gldm_DependenceNonUniformity	0.012	0.000	
wavelet_HHL_glrlm_GrayLevelNonUniformity	−0.005	0.004	
wavelet_HHL_glszm_ZoneVariance	0.000	0.462	
wavelet_LLL_glcm_Idmn	−12.578	0.025	
**Linear Regression of the Textural Features Extracted** **by** **VIBE_FA30 with Respect to the Recurrence Presence**	** Coefficients **	*** p *** ** value **	*** p *** ** value **
Intercept	−0.018	0.966	0.000
original_glszm_ZonePercentage	0.852	0.540	
wavelet_HLL_glcm_Correlation	−0.464	0.684	
wavelet_LHL_glcm_Correlation	5.324	0.001	
wavelet_LHL_glcm_SumEntropy	−0.243	0.474	
wavelet_LHL_glcm_Imc2	v2.956	0.037	
wavelet_LHL_glcm_ClusterTendency	−0.014	0.891	
wavelet_HLH_glcm_Correlation	2.266	0.341	
wavelet_HHL_glrlm_HighGrayLevelRunEmphasis	0.033	0.010	

## Data Availability

Data and material are available at https://zenodo.org/record/6299754#.Yh-ezOjMK3A.
